# Smartphone-RCCT: an online repository of randomized controlled clinical trials of smartphone applications for chronic conditions

**DOI:** 10.1186/s13063-022-06849-x

**Published:** 2022-10-27

**Authors:** Jürgen Barth, Jiani Wang, Jesus Lopez-Alcalde, Christoph Kramm, Daniel Pach, Noelia Álvarez-Díaz, Eulàlia Grifol-Clar, Claudia M. Witt

**Affiliations:** 1 Institute for Complementary and Integrative Medicine, University Hospital Zurich and University of Zurich, Zurich, Switzerland; 2 Charité – Universitätsmedizin Berlin, corporate member of Freie Universität Berlin and Humboldt-Universität zu Berlin, Institute of Social Medicine, Epidemiology and Health Economics, Berlin, Germany; 3 Faculty of Medicine, Universidad Francisco de Vitoria, Pozuelo de Alarcón, Spain; 4 Instituto Ramón y Cajal de Investigación Sanitaria (IRYCIS), Unidad de bioestadística clínica, Hospital Universitario Ramón y Cajal, (CIBERESP), Madrid, Spain; 5 Medical Library, Hospital Universitario Ramón y Cajal, IRYCIS, Madrid, Spain; 6 Medical Library, Hospital Universitario Fundación Alcorcón, Madrid, Spain; 7 Program for Integrative Medicine, University of Maryland School of Medicine, Baltimore, MD USA

**Keywords:** mHealth, eHealth, Smartphone, Database, App, Chronic conditions, Chronic diseases

## Abstract

**Background:**

Chronic health conditions have a big impact on disability, morbidity, and mortality worldwide. Smartphone health applications (apps) can improve the health of patients with chronic conditions and enhance the quality and efficiency of healthcare. The number of randomized controlled trials (RCTs) of smartphone health apps is increasing, but a collection of the available evidence into a single database is still missing. The purpose of this study is to describe *Smartphone-RCCT*, which is an in-progress database of RCTs of smartphone apps for chronic conditions.

**Methods:**

For a study to be included in the database, the following criteria had to be met: (a) RCT published in a peer-reviewed journal; (b) population: adult study participants with one or several chronic conditions that represent the main health problem addressed by the study intervention; (c) intervention: smartphone health app used by the patient; (d) comparator: any control condition; (e) outcomes: any patient-reported health outcome (studies exclusively measuring the patients’ knowledge about the chronic conditions or their satisfaction with the smartphone app were excluded); (f) sample size: at least 15 participants per study arm. We searched in electronic databases and other resources to identify relevant studies. Two reviewers selected the studies and extracted data independently. Annual updates are planned.

**Results:**

The proposed database is called Smartphone-RCCT, an open-access repository collecting bibliographic references and important characteristics of RCTs of smartphone apps for chronic conditions. The database is available for free in Open Science Framework (OSF): https://osf.io/nxerf/. To date, it includes 70 trials. Their references can be exported to standard reference management software and the extracted data is available in a Microsoft Excel file.

**Conclusions:**

Smartphone-RCCT is the first systematic open-access database collecting peer-reviewed publications of RCTs of smartphone apps for patients with chronic conditions. The database accelerates the delivery of evidence-based information in a dynamic research field. It represents an essential resource for different stakeholders, such as professionals working in evidence synthesis, meta-epidemiological studies, or planning an RCT.

**Supplementary Information:**

The online version contains supplementary material available at 10.1186/s13063-022-06849-x.

## Background

Chronic health conditions require ongoing management for years or decades [1]. They cover a wide range of health problems beyond the traditional concept of noncommunicable diseases [1]. Chronic health conditions have a long duration and a generally slow progression [2] and require a complex response from the healthcare system over an extended period [1].

Chronic conditions are frequent and affect people worldwide, regardless of their level of income, age, and sex. Approximately one-third of all adults worldwide suffer from more than one chronic condition [3], and close to 75% of older adults in high-income countries present multiple chronic conditions. Moreover, the incidence and prevalence of chronic conditions and multiple chronic conditions will rise dramatically in the future because of the aging population [3–5].

Chronic conditions have a tremendous impact at the individual and societal levels. They reduce the quality of life and cause premature deaths, representing the leading cause of death and disability in adults worldwide [6]. Chronic conditions also pose a significant economic burden on individuals and their households [7]. Finally, the enormous financial healthcare costs caused by chronic conditions will increase in the coming years, threatening to overwhelm health systems worldwide [1, 8, 9].

### The rationale for a repository of randomized clinical trials (RCTs) of smartphone apps for chronic conditions

Mobile Health (mHealth) is the medical and public health practice supported by mobile devices [10, 11]. Examples of mobile devices are mobile phones (such as smartphones), tablets, personal digital assistants (PDAs), and wireless devices (such as smartwatches and patient-monitoring devices) [11, 12]. Smartphones are an integral part of the daily life of citizens worldwide (penetration rate > 75%) [13], and their number is continuously increasing [14]. A smartphone application (app) is a software or program designed for a mobile device.

mHealth is a fast-developing field gaining popularity among citizens, patients, healthcare professionals, and for-profit institutions [15–18]. This interest explains how there are now more than 325,000 health apps available [19]. The proliferation of health apps will modify the interaction between the patient and the healthcare system [20]. mHealth apps can help in numerous clinical practice scenarios [18], such as in support of clinical diagnosis and decision-making or as standalone digital therapeutics, which is the focus of this paper. An example of digital therapeutics is the digitalization of cognitive-behavioral therapy (CBT), a classic form of therapy that is now also offered digitally [18]. Also, mHealth has the potential to increase health promotion, prevention, and early diagnosis, which in turn might prevent or delay the onset of chronic conditions [1].

mHealth can empower citizens by facilitating the self-management of their health and by supporting personalized health and care [12, 21, 22]. Furthermore, mHealth is expected to improve the quality of life for individuals in chronic conditions, which is usually low [8]; patients with chronic conditions often go untreated [1], and mHealth is expected to improve professional and patient adherence to the treatment by facilitating the patient-health professional interaction. Finally, mHealth has the potential to increase the efficiency and sustainability of healthcare [23]. For example, routinely scheduled appointments at a clinic can be reduced, liberating the professionals to focus on patients with chronic conditions requiring urgent attention [1]. For these reasons, governments and research funding institutions worldwide recognize mHealth (and smartphone health apps) as a strategic area to overcome the burden of chronic conditions.

High-quality evidence is still needed to better understand if mHealth can be used to add value to clinical care [18]. Besides, there is still room for improvement in the smartphone health apps’ accuracy, reliability, and effectiveness [24–26]. Finally, their use raises additional concerns, such as the security and privacy of individual health data, the lack of clinical guidelines for their application in clinical practice, or the equal access to healthcare via mHealth by the population [22]. As RCTs are the mainstay of evidence-based medicine, mHealth app effectiveness and safety should be continuously demonstrated in rigorous evaluations based on RCTs.

### Purpose and utilities of the Smartphone-RCCT database

Smartphone-RCCT stands for *Smartphone-Randomised Chronic Conditions Trials*. It is a repository of bibliographic references and important characteristics of RCTs of smartphone apps used by patients to improve or manage their chronic conditions. The database is freely available in Open Science Framework (OSF): https://osf.io/nxerf/ [27].

To our knowledge, Smartphone-RCCT is the first centralized repository collecting RCTs evaluating the effects of smartphone health apps. There are other databases of smartphone applications [28–31], but they do not focus on RCTs and chronic conditions. Besides, as this article will detail, our repository will collect studies measuring health outcomes in patients, which is critical information for supporting clinical decisions.

Smartphone-RCCT is designed to be a systematic, updated, and open resource that provides an overview of peer-reviewed publications of RCTs evaluating the effects (effectiveness and safety) of smartphone apps for patients with chronic conditions. The database aims to ensure that its users have access to the maximum number of relevant RCTs in this field.

Smartphone-RCCT has three main utilities. First, it provides an overview of the existing RCTs of smartphone apps for chronic conditions. To maintain a database with RCTs is essential as RCTs are deemed to yield the most reliable estimates about the effects of healthcare interventions [32–34]. In this line, developers of clinical guidelines consider well-done systematic reviews of RCTs as the best evidence for answering questions on healthcare interventions [34–36]. Moreover, the number of RCTs on smartphone interventions increases, so it will be challenging to maintain an overview. Thus, identifying all relevant RCTs and maintaining a database are critical steps in supporting informed healthcare decisions [32, 35, 37]. Second, Smartphone-RCCT classifies the included RCTs into specific categories. Users can quickly access studies according to relevant criteria, such as the type of chronic condition or health outcome. Thus, the database provides an intuitive search environment for study identification and allows identifying research gaps to inform further research needs. Third, the user can use standard reference management software to import the content of *Smartphone-RCCT.*

This paper presents the Smartphone-RCCT database and its development methods. The paper further describes the characteristics of the database and the currently available data.

## Methods

### Project team

**Fig. 1:**
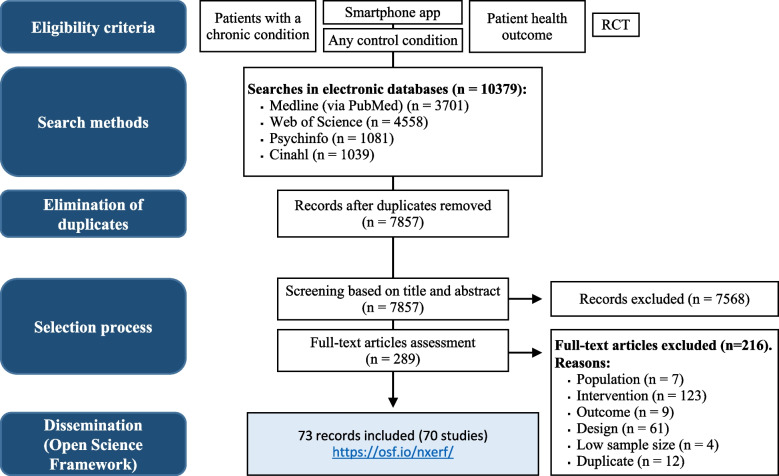
Flow diagram

*The Institute for Complementary and Integrative Medicine* [38], which is part of the *University Hospital Zurich* (Zurich, Switzerland), and *Cochrane Complementary Medicine Switzerland* launched Smartphone-RCCT. The team is closely connected to Cochrane and has a solid background to perform searches, screening, and data extraction efficiently. Experienced librarians will conduct searches for the update of the database. We will follow the methods described below to create and maintain the database. Figure 1 summarizes the process in a flow diagram.

### Eligibility criteria to include studies in the database

So far, the following eligibility criteria have been used for inclusion in the database. We reserve the right to modify the criteria in the future if it seems justified to improve the suitability of the database for researchers, since topics in digital health might change over time. Any change in the eligibility criteria would then be described transparently and in detail.

#### Type of publications

The database includes reports of studies published as an article after 2005 (2 years before the launch of the first iPhone in 2007). The database does not include reports of theses, conference presentations, or ongoing trials. It is not intended to search for preprint archives, such as those disseminated in *bioRxiv* or *medRxiv*. There are no restrictions concerning the language of publication. All studies in the database evaluate the effectiveness or safety of smartphone apps used by adults to improve or manage their chronic condition.

#### Study designs

RCTs are studies in which the participants were assigned to a study arm prospectively using a random allocation process (e.g., random number generation or coin flips). Pilot studies with less than 15 participants per arm are not eligible. Non-randomized studies (quasi-RCTs, non-randomized controlled clinical trials, or observational studies) are excluded. We include those studies that were reported as randomized but for which the sequence generation method is unclear and for which we cannot obtain clarification from the authors.

#### Types of participants

Studies are included if they included adults (18 years old or older) with one or several chronic conditions. The chronic condition must represent the main health problem investigated in the study. For example, an RCT of a smartphone app to improve the uptake of antibiotic treatment in patients with community-acquired pneumonia would be excluded, even if the patients also had a chronic condition disease, such as diabetes.

According to the WHO proposal, chronic conditions are defined as conditions “requiring ongoing management over a period of years or decades” [1]. To be considered chronic, a condition needs to be chronic per se (i.e., diabetes), or the symptoms need to have lasted at least 3 months [39], e.g., chronic low back pain. If the duration of the condition is not reported, but the condition is usually considered chronic, we classify the study as eligible.

Studies with patients with mental disorders are eligible if the symptom duration is expected to last more than 3 months (i.e., depression, anxiety disorders). Temporary mental disorders are excluded (i.e., adjustment disorder, intoxication).

Studies focusing on the prevention or management of overweight [body mass index (BMI) below 30] or obesity (BMI of 30 or higher) are included if the overweight/obesity prevention or management aims at improving a chronic condition (e.g., a smartphone application to reduce overweight in diabetic patients). However, studies with healthy individuals and individuals with a risk factor that is not an ICD diagnosis are excluded.

To be eligible, the health problem needs to be included in the WHO International Classification of Diseases (ICD) (10th Revision) or the Diagnostic and Statistical Manual for Mental Disorders (DSM). Additional file 1 details a list of eligible chronic conditions (not exhaustive). Studies that include healthy participants exclusively or a population that might be a mix of healthy subjects and patients are excluded. Thus, the database excludes studies of interventions for the primary prevention of chronic conditions.

#### Types of interventions

Smartphone-based app interventions of any duration when used by patients with a chronic condition to manage that chronic condition. We consider the following definitions:

- Smartphone: a cellular phone, also called a mobile phone, equipped with various additional features, mobile operating systems, multimedia functionality, Internet access, and basic functionalities (text messaging, voice calls) [40]
- Smartphone app: software or program designed for a smartphone device

The main intervention evaluated in the study must be a smartphone app. Thus, we exclude studies that use other types of mHealth interventions, such as primarily Web-based interventions or interventions delivered via tablets, PDAs, monitoring devices (such as devices collecting exercise information), smartwatches, or other mobile devices. When the smartphone app intervention is delivered with other co-interventions, such as face-to-face interaction with a healthcare provider, the study is eligible. However, co-interventions must be limited in time, and the smartphone app intervention must be the main intervention evaluated in the study. Consequently, we exclude studies in which the smartphone app is just a co-intervention of comprehensive face-to-face intervention. We exclude smartphone apps used exclusively for diagnostic purposes. However, if the app has a screening functionality, evaluated as part of an intervention trial, the study is eligible.

To our knowledge, there is no standardized classification for mHealth interventions yet; our decisions concerning the eligibility of the interventions are guided by the NICE classification of mHealth interventions [41] (Additional file 2).

#### Types of comparators

We include trials with any control condition, whether active or inactive. However, if the comparator consists of only a minor adaptation of the app intervention of the active treatment condition (e.g., the duration of the intervention or the design of the smartphone application), the study is excluded.

#### Types of outcome measures

The trial must measure health outcomes in patients (no specific measurement tool or timeframe is mandatory), such as HbA1c levels, quality of life, depression, fatigue, or exercise behavior. However, studies exclusively measuring outcomes concerning the knowledge of the patient about the chronic condition or his/her satisfaction with the smartphone application are excluded. We also exclude studies only reporting results not measured in patients, such as the impact of the chronic condition on the patient’s relatives or the healthcare system, such as costs or economic implications.

### Identification of the studies: search methods

The database in its current state includes the results of initial searches in MEDLINE, Web of Science, PsycInfo, and CINAHL. We plan to perform periodical searches (once per year) in electronic databases and other resources. Two experienced librarians have designed the search strategies. The librarians will be involved in the updates of the database, which will search the following sources.

#### Searching electronic databases

We will search the following electronic databases.

- MEDLINE (via Ovid)
- Embase (via Elsevier)
- CENTRAL (via Wiley)
- PsycInfo (via Ebsco)
- CINAHL (via Ebsco)

Additional file 3 presents the search strategies that will be used in each electronic database. The smartphone terms were adapted from the search strings considered in two Cochrane systematic reviews [42, 43]. The search strategy does not consider terms related to chronic conditions because chronic conditions cover many health problems (Additional file 1). Moreover, some conditions are inconsistently labeled as chronic. To avoid the inadequate use of terms in our search strategy and, thus, miss eligible studies, we implement a broader search and omit chronic conditions terms. Thus, we assess the eligibility of all the RCTs of smartphone apps.

For identifying RCTs, we use the methodological filters developed by the Scottish Intercollegiate Guidelines Network (SIGN) [44]. There are no restrictions concerning the language of publication. We may modify the search strategy in future updates if we detect opportunities for improvement.

#### Searching other resources

We will consult *Epistemonikos* [45] to identify systematic reviews of smartphone interventions. In turn, we will screen the references of these systematic reviews to identify additional RCTs eligible for our database.

### Study selection

We developed a coding manual to implement the selection process. Two people (KE and JW) piloted this coding manual with 48 titles and abstracts. The decisions were checked by a third person (JB). We may modify the manual if we detect opportunities for improvement.

We imported all results of the searches into Endnote X9 [46] and deleted duplicates. We transferred the unique records to Rayyan QCRI [47] and used this software to implement the selection process. Two reviewers independently screened the titles and abstracts. Each record was classified as “Included,” “Excluded,” or “Maybe included.” We obtained the full text of those records defined as “Included” or “Maybe included.” Two reviewers independently assessed the full texts for inclusion. We classified each full text as “Included” or “Excluded.” At all the stages of the selection process, we tried to resolve disagreements by consensus between the initial raters. If disagreements could not be solved, a third person was consulted. If information is insufficient to determine the eligibility, the study authors were contacted for clarification. We were not blinded to the publication details during the selection of the studies. If there were multiple reports of the same study, they were collated so that each study, rather than the report, was the unit of interest in the database. We chose the most complete and recent report as the main source for each study.

### Data extraction procedure

We created a Microsoft Excel form to extract the data for each study included in the database and piloted the form for usability. Two reviewers independently performed the data extraction for each study. Any discrepancy was solved by consensus. If disagreements could not be resolved, a third person intervened. We planned to contact the study authors for clarification or missing data. If we could not clarify the issues, we described the corresponding field as “unclear.” If errors are reported, or the study authors ask for corrections of their own material, we will amend the data.

## Results

### Overview of the characteristics of the database

**Fig. 2:**
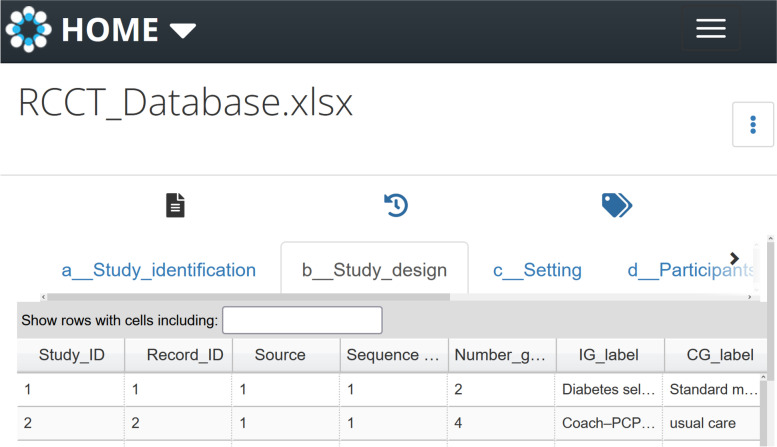
Screenshot of the database

The first version of Smartphone-RCCT is available online in OSF (https://osf.io/nxerf/) [27]. Figure 2 shows a screenshot of one sheet of the database.

On 19 April 2022, it included 70 trials and 73 records (two studies were reported in three and two articles, respectively). These results were based on a search strategy executed in April 2019. The extracted data is available in a Microsoft Excel file. Besides, the references of the database studies are provided as a file compatible for importation to standard reference management software, such as Endnote or Mendeley*.* For each study, the following information is available (see the full codebook in Additional file 4). Those aspects marked with * will be implemented in future updates of the database. Additional file 5 explains how to reach the database and its structure in OSF.

#### Study identification

Each study included in the database is identified with the following information: (1) study identification number; (2) record identification number (if several reports refer to the same study, the most complete and up-to-date report is defined as the “primary report”; the remaining reports will be classified as “secondary reports”); (3) the first and the last authors of the primary report; (3) year of publication; (4) language of publication; (5) reference (citation); (6) article identification number, such as the digital object identifier (doi); and (7) the date when the record was incorporated to the database. This database is “study-based,” so all the references to the same study are identified with the same “study identification number.” The export files contain all the references, so more records than studies may be generated. To group all the references linked to the same study, sort by the column “Study identification number” in the excel file.

#### Study design

Critical information of the design of each included study is displayed, covering the following aspects: (1) sequence generation method; (2) unit of allocation*; (3) type of trial* (such as parallel, factorial, or crossover); and (4) the number of study arms. The type of intervention and the control group are labeled to facilitate their identification.

#### Study setting

The database contains the following information regarding the setting of each included study: (1) country where the study was carried out; (2) channel/s that were used to recruit the study participants; (3) setting from which the study participants were recruited (inpatients, outpatients, or both)*; and (4) the efficacy-effectiveness nature of the trial according to the RITES (*Rating of Included Trials on the Efficacy-effectiveness Spectrum*) tool [48].

#### Participant’s characteristics

The database illustrates the basal characteristics of the study participants particularly the following features: (1) sex*, (2) availability of results disaggregated by sex*, (3) gender*, (4) availability of results disaggregated by gender*, (5) age*, (6) educational level*, (7) socioeconomic level*, and (8) chronic health condition addressed in the study (preferably selected from the list in Additional file 1).

#### Intervention details

The database covers critical information of the experimental interventions, such as the following: (1) app developer*: name and type (non-/for-profit organization, university, or another public research institution, or others); (2) the start date of the intervention*; and (3) theoretical framework for the intervention.

The database classifies the intervention according to the implemented behavior change techniques (BCT) as follows: (1) patient education to follow the BCT intervention, (2) feedback and monitoring of the BCT intervention, (3) goals and planning of the intervention, (4) communication between users of the app, and (5) intervention attempts to reduce negative emotions, stress, and prompts or cues.

The context in which the app intervention was delivered is also extracted, such as if there was an app and face-to-face therapy or the therapy included an app and virtual contact with the therapist.

Finally, the database describes the planned dose and intensity of the intervention (number of sessions and duration in weeks) and the expected impact of the co-interventions on the patient’s health, including training/support. Also, the application’s cost to the study participants and their financial compensation for participating in the trial are detailed.

#### Comparator details

The database describes the control condition* of each included study according to the Gold et al. classification [49], which includes the following categories: (1) active comparator, (2) minimal treatment control, (3) non-specific factors component control (also known as psychological placebo), (4) no-treatment control, (5) patients’ choice, (6) placebo pill, (7) specific factors component control, (8) treatment as usual, (9) waitlist control, and (99) unclear (Additional file 4 details each category).

#### Outcomes

The database includes the outcomes that were reported in each study, e.g., patient-reported health, quality of life, symptom severity, depression, anxiety, felt stress, physical activity, weight, health/risk behaviors, measures for disease management of disorder (diabetes, hypertension, etc.), motivation, pain, mindfulness (self-assessment), satisfaction with the intervention, or adherence to the app intervention and the treatment (attrition, drop-outs).

## Discussion

### Principal results

This paper introduces *Smartphone-RCCT*, an open-access bibliographic database with RCTs of smartphone apps for patients with chronic health conditions. This database will cover important information of those RCTs to facilitate systematic reviews in this field. The database is freely available as an open resource in OSF (https://osf.io/nxerf/) [27] and contains 70 trials (19 April 2022).

The database compiles critical information of the included studies, such as the study design and setting, the study participants’ characteristics, the experimental intervention, and the control intervention, as well as the reported participants’ health outcomes. This overview of the available RCTs can be useful for different stakeholders. In particular, the database will be valuable to professionals working in evidence synthesis (such as systematic reviews), researchers developing meta-epidemiological studies, and trialists and funding agents planning to start an RCT. Our project will also align with other initiatives attempting to identify RCTs, such as *CENTRAL*, and with institutions and researchers aiming to disseminate the results of their RCTs.

### Potential users of the database

Several stakeholders will benefit from the use of *Smartphone-RCCT*:

1. Evidence synthesis professionals, such as systematic reviewers. The database will help identify studies, a time-consuming step in systematic reviews of app research, a fast-moving field for which delays of 2 years between the systematic reviews searches and the analysis are common [50]. Besides, heterogeneous search strategies in systematic reviews of mHealth interventions can yield different included studies and heterogeneous findings among reviews with similar inclusion criteria [50, 51]. Our database can help to reduce this heterogeneity. On the other hand, we acknowledge that our database will not replace other resources to identify RCTs in systematic reviews, such as CENTRAL, MEDLINE, or Embase. However, we still think our repository will serve as a complementary source for the routine searches performed in systematic reviews. It can be a valuable tool that the reviewers can use to check that they missed no eligible trial.
2. Researchers developing meta-epidemiological research [52] of RCTs of smartphone health apps, for example, to identify app effect moderators. Our database provides a collection of RCTs ready to be analyzed (the search strategies of health app intervention studies often generate about 10,000 references, of which only a small part will be eligible [53]).
3. Researchers and funding agents planning to start an RCT. The database can help to quickly identify the studies that have already been conducted on a specific clinical question. This information helps to decide whether further research is necessary and helpful. Thus, the database will contribute to avoiding research waste [54].
4. Other initiatives to identify RCTs. The Smartphone-RCCT team is part of Cochrane, so the records included in our database are ready to be shared with the *Cochrane Central Register of Controlled Trials* (CENTRAL) [55].
5. Institutions and researchers aiming to disseminate the results of app intervention studies. Smartphone-RCCT contributes to disseminating app intervention studies, for example, by facilitating their inclusion in systematic reviews or guidelines.

### Comparison with prior work

There are several initiatives collecting trials on different health conditions. An example is The Cochrane Central Register of Controlled Trials (CENTRAL) [53], which is the biggest register of trials in the world. This database has been demonstrated to be essential for systematic reviewers worldwide. Another well-known initiative is the Cuijpers et al. register of RCTs on psychotherapies for depression [54]. Till July 2019, this database has allowed the generation of 81 peer-reviewed papers [55]. However, the scope of this database is more limited than ours, as it focuses on RCTs of psychotherapies for depression. Besides, our database has important fields for future meta-epidemiological studies, such as the classification of the control condition [51].

### Limitations of the database

A limitation is the risk of missing studies with selective outcome reporting. As studies that did not report health outcomes measured in patients are excluded, those RCTs are consequently missed in the database, even if health outcomes in patients were measured but the results not reported.

On the other hand, we foresee that the reports will often lack a detailed description of critical aspects, such as the control intervention. In case of doubt, we will contact the study authors for clarification, but we may not succeed in obtaining the required information. However, we will register this lack of information and will highlight the inadequate reporting, which is an important finding of the project: the database will help to identify those aspects that need to be better described in the published literature.

Although we will try to find all the published RCTs on smartphone apps for patients with chronic conditions, we acknowledge that the published trials may not fully represent all the RCTs done due to publication bias. A limitation of the database is that gray literature is not searched (i.e., reports of theses, conference presentations, and preprint archives). Besides, we will not search for ongoing trials, which would help to identify those trials that were registered but whose results were not published.

Another limitation results from the impact of language bias: although we do not limit our search strategies by the language of publication, we only use North American and European electronic databases. Therefore, we do not search other important databases, such as those located in China. However, we will consult CENTRAL, which consists of records retrieved from numerous sources, and is not limited by the language of publication.

A final challenge is maintaining the database up to date, as the selection of the studies will be manual, that is, not based on a process automatically linking the database to new trials. As highlighted before, mHealth is a fast-developing field, and therefore, identifying all the trials in this field is challenging. To minimize this constraint, we will use the *Epistemonikos* database to select the RCTs included in smartphone app intervention reviews. At present, *Epistemonikos* is the largest database of systematic reviews: it uses a machine-learning approach to identify systematic reviews in ten bibliographic databases on a daily or weekly basis [56]. In turn, we will screen these systematic reviews to identify additional RCTs eligible for our database. This will allow an efficient selection process.

## Conclusions

Smartphone-RCCT is the first systematic open-access database that provides an overview and important characteristics of peer-reviewed publications of RCTs evaluating the effects (effectiveness and safety) of smartphone apps for patients with chronic conditions.

Smartphone-RCCT aims to ensure that there is open access to the maximum number of relevant RCTs in this field. Thus, the database will be useful to different stakeholders, such as professionals working in evidence synthesis (e.g., systematic reviewers and guideline developers), researchers developing meta-epidemiological studies, trialists and funding agents planning to start an RCT, other initiatives to identify RCTs, such as CENTRAL, and institutions and researchers aiming to disseminate the results of RCTs.

## Supplementary Information

**Additional file 1.** Chronic health conditions

**Additional file 2.** Classification of digital health technologies (DHTs)

**Additional file 3.** Search strategies

**Additional file 4.** Codebook

**Additional file 5.** Database structure in Open Science Framework

## Data Availability

The datasets generated during and/or analyzed during the current study are available in the Smartphone-RCCT repository [https://osf.io/nxerf/] [27].

## Abbreviations

RCT: Randomized controlled trial
