# Supplementary material for: Smartphone-RCCT: an online repository of randomized controlled clinical trials of smartphone applications for chronic conditions
Source: Trials. 2022 Oct 27;23:909. doi: 10.1186/s13063-022-06849-x (PMC9615349; doi:10.1186/s13063-022-06849-x)
Supplement: Supplementary file 1 — Additional file 1. Chronic health conditions [file 13063_2022_6849_MOESM1_ESM.docx]

**Additional file 1.** **Chronic health conditions**

| a. Conventional non-communicable diseases | ▪ Cancer. Examples: breast cancer, lung cancer, prostate cancer, or colorectal cancer  ▪ Cardiovascular disease. Examples: Heart disease (such as coronary heart disease or heart failure), stroke or hypertension.  ▪ Chronic respiratory disease. Examples: chronic obstructive pulmonary disease, asthma, allergic rhinitis, or obstructive sleep apnea  ▪ Mental disorders. Examples: depression, schizophrenia, bipolar disorder, phobias or Post-traumatic Stress Disorder.  ▪ Dementia: Examples: Alzheimer’s disease or Parkinson's disease  ▪ Diabetes  ▪ Gastrointestinal disease. Examples: inflammatory bowel disease, or permanent stoma  ▪ Cirrhosis  ▪ Phobias  ▪ Urinary incontinence  ▪ Arthritis  ▪ Psoriasis  ▪ Chronic renal disease  ▪ Chronic pain  ▪ Insomnia  ▪ Other |
| --- | --- |
| b. Communicable diseases that have become controllable health problems for many years | ▪ HIV (Human immunodeficiency virus) and AIDS (acquired immunodeficiency syndrome)  ▪ Chronic hepatitis infection  ▪ Other |
| c. Disabilities and impairments not defined as diseases | ▪ Visual impairment or blindness  ▪ Hearing impairment and deafness  ▪ Musculoskeletal disorders. Examples: non-specific chronic back pain or frozen shoulder (shoulder pain for at least one month)  ▪ Other |
| d. Genetic disorders | ▪ Down syndrome  ▪ Cystic fibrosis  ▪ Other |
