# Supplementary material for: Smartphone-RCCT: an online repository of randomized controlled clinical trials of smartphone applications for chronic conditions
Source: Trials. 2022 Oct 27;23:909. doi: 10.1186/s13063-022-06849-x (PMC9615349; doi:10.1186/s13063-022-06849-x)
Supplement: Supplementary file 2 — Additional file 2. Classification of digital health technologies (DHTs) [file 13063_2022_6849_MOESM2_ESM.docx]

**Additional file 2. Classification of digital health technologies (DHTs)**

There are several approaches to categorize mHealth application. This classification is based on the National Institute for Health and Care Excellence (NICE) proposal to classify digital health technologies (DHTs) (1), which categorizes DHTs by their function. This system allows allocating each DHT into an evidence tier that is proportionate to the potential risk to users presented by the DHT. Following we detail the functional classification of DHTs with the corresponding evidence tiers.

| **Evidence tier** | **Functional classification** | **Explanation** | **Examples** | **Eligibility for our database** |
| --- | --- | --- | --- | --- |
|  |  |  |  |  |
| **Tier 1: DHTs with potential system benefits but no direct user benefits** | “System service” | ▪ DHTs to improve the system efficiency.  ▪ They are unlikely to have direct and measurable individual patient outcomes. | ▪ Electronic health record platforms | Excluded |
|  |  |  |  |  |
| **Tier 2: DHTs which help users to understand healthy living and illnesses but with unlikely measurable user outcomes** | “Inform” | ▪ DHTs that provide information and resources to the public or patients.  ▪ Can include information about healthy living or specific health conditions. | ▪ Apps providing advice for healthy lifestyles, such as recipes.  ▪ Apps describing a condition and its treatment. | Excluded |
|  | “Simple monitoring” | ▪ DHTs that allow users recording health parameters to create health diaries.  ▪ The information is not shared with others. | ▪ Health tracking information apps, such as fitness wearables or symptom or mood diaries. | Excluded |
|  | “Communicate” | ▪ DHTs that allow a bidirectional communication between users and professionals, carers, third-party organizations or peers.  ▪ Clinical advice is provided by a professional using the DHT, not by the DHT itself. | ▪ Platforms for communication with the professional, such as:  a) Healthcare telephone helplines: health care advice and triage provided by trained personnel and pre-recorded messages; accessible on mobile phones or fixed lines(2)  b) Emergency toll-free  telephone services: free telephone hotlines for health emergencies provided by trained personnel and pre-recorded messages and linked to response systems; accessible on mobile phones or fixed lines (2). | Excluded |
|  |  |  |  |  |
| **Tier 3a: DHTs for preventing and managing diseases. They may be used alongside treatment and will likely have measurable user benefits** | 3a1. “Preventative behavior change” | ▪ DHTs designed to change user behavior related to health issues.  ▪ These DHTs are prescribed by a professional. | ▪ App for smoking cessation  ▪ App used as part of weight loss programme | Included |
|  | 3a2. “Self-manage” | ▪ DHTs designed to help people with a diagnosed condition to manage their health.  ▪ These DHTs allow users to record, and optionally to send, data to a healthcare professional to improve management of their condition. | ▪ App that provides reminders to attend appointments  ▪ App that provides reminders to increase medication adherence | Included |
|  |  |  |  |  |
| **Tier 3b: DHTs with measurable user benefits, including tools used for treatment and diagnosis, as well as those influencing clinical management through active monitoring or calculation.**  It is possible DHTs in this tier will qualify as medical devices | 3b1. “Treat” | ▪ DHTs that provide treatment for a diagnosed condition.  ▪ DHTs that guide treatment decisions. | ▪ Apps for treating mental health or other conditions, for example, CBT for anxiety.  ▪ Clinician-facing apps that advise on treatments in certain situations. | Included |
|  | 3b2. “Active monitoring” | ▪ DHTs that automatically record information and transmits the data to a professional, carer or third-party organization, to inform clinical management decisions.  ▪ These DHTs have no input from the user. | ▪ DHTs linked to devices such as implants, sensors worn on the body or in the home.  ▪ Data are automatically transmitted through the DHT for remote monitoring. | Excluded |
|  | 3b3. “Calculate” | ▪ Tools that perform clinical calculations that are likely to affect clinical care decisions.  ▪ DHTs for use by professionals or users to calculate parameters pertaining to care | ▪ Early warning system software | Included if it requires an active involvement by the patient |
|  | 3b4. “Diagnose” | ▪ DHTs that use data to diagnose a condition in a patient, or to guide a diagnostic decision made by a healthcare professional |  | Excluded |
|  |  |  |  |  |

**References**

1. National Institute for Health and Care Excellence (NICE). Evidence standards framework for digital health technologies 2019 [cited 2020 15 March]. Available from: <https://www.nice.org.uk/Media/Default/About/what-we-do/our-programmes/evidence-standards-framework/digital-evidence-standards-framework.pdf>.

2. World Health Organization. Global diffusion of eHealth: making universal health coverage achievable. Report of the third global survey on eHealth. Geneva; 2016. Report No.: Licence: CC BY-NC-SA 3.0 IGO.
