# Supplementary material for: Smartphone-RCCT: an online repository of randomized controlled clinical trials of smartphone applications for chronic conditions
Source: Trials. 2022 Oct 27;23:909. doi: 10.1186/s13063-022-06849-x (PMC9615349; doi:10.1186/s13063-022-06849-x)
Supplement: Supplementary file 3 — Additional file 3. Search strategies [file 13063_2022_6849_MOESM3_ESM.docx]

**Appendix 3. Search strategies**

| **1. Search strategy in Medline (via Ovid)** |
| --- |
|  |
| Database: Ovid MEDLINE(R) ALL / PubMed(R) <1946 to Present>  1 Smartphone/  2 Cell Phone/  3 (smart-phone? or smartphone?).tw.  4 ((cell? or mobile?) adj3 phone?).tw.  5 android.tw.  6 iphone.tw.  7 (App? adj3 (Smartphone? or Smart-phone? or mobile? or phone?)).tw.  8 Telemedicine/  9 (tele-medicine or telemedicine or tele-health or telehealth).tw.  10 (mhealth or m-health or "m health" or mobile health).tw.  11 or/1-10  12 Randomized Controlled Trials as Topic/  13 Randomized Controlled Trial/  14 Random Allocation/  15 Double-Blind Method/  16 Single-Blind Method/  17 Clinical Trial/  18 clinical trial, phase i.pt.  19 clinical trial, phase ii.pt.  20 clinical trial, phase iii.pt.  21 clinical trial, phase iv.pt.  22 controlled clinical trial.pt.  23 randomized controlled trial.pt.  24 multicenter study.pt.  25 clinical trial.pt.  26 exp Clinical Trials as Topic/  27 (clinical adj trial$).tw.  28 ((singl$ or doubl$ or treb$ or tripl$) adj (blind$3 or mask$3)).tw.  29 Placebos/  30 placebo$.tw.  31 randomly allocated.tw.  32 (allocated adj2 random$).tw.  33 or/12-32  34 11 and 33  35 34 and 2005:2020.(sa_year). |

| **2. Search strategy in Embase (Elsevier)** |
| --- |
|  |
| #28. #11 AND #27 AND [embase]/lim AND [2005-2020]/py  #27. #12 OR #13 OR #14 OR #15 OR #16 OR #17 OR #18 OR #19 OR #20 OR #21 OR #22 OR #23 OR #24 OR #25 OR #26  #26. placebo$:ab,ti  #25. (treble OR triple) NEAR/3 blind*  #24. 'single blind*':ab,ti  #23. 'double blind*':ab,ti  #22. (random* NEAR/2 allocat*):ab,ti  #21. rct:ab,ti  #20. randomi?ed:ab,ti AND controlled:ab,ti AND trial$:ab,ti  #19. 'placebo'/de  #18. 'crossover procedure'/de  #17. 'double blind procedure'/de  #16. 'randomization'/de  #15. 'phase 4 clinical trial'/de  #14. 'phase 3 clinical trial'/de  #13. 'multicenter study'/de  #12. 'clinical trial'/de  #11. #1 OR #2 OR #3 OR #4 OR #5 OR #6 OR #7 OR #8 OR #9 OR #10  #10. mhealth:ab,ti OR 'm health':ab,ti OR 'mobile health':ab,ti  #9. 'tele medicine':ab,ti OR telemedicine:ab,ti OR 'tele health':ab,ti OR telehealth:ab,ti  #8. 'telemedicine'/de  #7. app* NEAR/3 (smartphone* OR 'smart phone*' OR mobile* OR phone*)  #6. iphone:ab,ti  #5. android:ab,ti  #4. ((cell* OR mobile$) NEAR/3 phone$):ab,ti  #3. 'smart phone$':ab,ti OR smartphone$:ab,ti  #2. 'mobile phone'/de  #1. 'smartphone'/de |

| **3. Search strategy in Cochrane CENTRAL (Wiley)** |
| --- |
|  |
| #1 MeSH descriptor: [Smartphone] this term only  #2 MeSH descriptor: [Cell Phone] this term only  #3 (App? NEAR/3 (Smartphone? or Smart-phone? or mobile? or phone?)):ti,ab,kw  #4 MeSH descriptor: [Telemedicine] this term only  #5 (tele-medicine or telemedicine or tele-health or telehealth):ti,ab,kw  #6 {OR #1-#5} with Publication Year from 2005 to 2020, in Trials |

| **4. Search strategy in APA PsycInfo (Ebsco)** |
| --- |
|  |
| S1 DE "Smartphones"  S2 DE "Mobile Phones"  S3 TX smart-phone* or smartphone*  S4 TX (cell* or mobile*) n3 phone*  S5 TX android  S6 TX iphone  S7 TX App* n3 (smartphone* or smart-phone or mobile* or phone*)  S8 DE "Telemedicine"  S9 TX tele-medicine or telemedicine or tele-health or telehealth  S10 TX mhealth or m-health or "mhealth" or mobile health  S11 S1 OR S2 OR S3 OR S4 OR S5 OR S6 OR S7 OR S8 OR S9 OR S10 (23,383)  S12 DE "Clinical Trials" OR DE "Randomized Controlled Trials"  S13 PT clinical trial  S14 TX clinic* n1 trial*  S15 TX ( (singl* n1 blind*) or (singl* n1 mask*) ) or ( (doubl* n1 blind*) or (doubl* n1 mask*) ) or ( (tripl* n1 blind*) or (tripl* n1 mask*) ) or ( (trebl* n1 blind*) or (trebl* n1 mask*) )  S16 TX randomi* control* trial*  S17 DE "Random Sampling"  S18 TX random* allocat*  S19 TX placebo*  S20 DE "Placebo"  S21 DE "Quantitative Methods"  S22 TX allocat* random*  S23 S12 OR S13 OR S14 OR S15 OR S16 OR S17 OR S18 OR S19 OR S20 OR S21 OR S22  S24 S11 AND S23  S25 S11 AND S23 Limiters - Publication Year: 2005-2020 |

| **5. Search strategy in CINAHL (Ebsco)** |
| --- |
|  |
| S1 (MH "Smartphone")  S2 (MH "Cellular Phone")  S3 TI (smart-phone* or smartphone*) or AB (smart-phone* or smartphone*)  S4 TI ("cell phone" or "mobile phone") or AB ("cell phone" or "mobile phone")  S5 TI android or AB android  S6 TI iphone or AB iphone  S7 TI App* n1 (smartphone* or smart-phone* or mobile* or phone*) or AB App* n1 (smartphone* or smart-phone* or mobile* or phone*)  S8 (MH "Telemedicine")  S9 TI (tele-medicine or telemedicine or tele-health or telehealth) or AB (tele-medicine or telemedicine or tele-health or telehealth)  S10 TI (mhealth or m-health or "mhealth" or mobile health) or AB (mhealth or m-health or "mhealth" or mobile health)  S11 (S1 OR S2 OR S3 OR S4 OR S5 OR S6 OR S7 OR S8 OR S9 OR S10) (34,280)  S12 (MH "Clinical Trials+")  S13 PT clinical trial  S14 TX clinic* n1 trial*  S15 TX ( (singl* n1 blind*) or (singl* n1 mask*) ) or ( (doubl* n1 blind*) or (doubl* n1 mask*) ) or ( (tripl* n1 blind*) or (tripl* n1 mask*) ) or ( (trebl* n1 blind*) or (trebl* n1 mask*) )  S16 TX randomi* control* trial*  S17 (MH "Random Assignment")  S18 TX random* allocat*  S19 TX placebo*  S20 (MH "Placebos")  S21 (MH "Quantitative Studies")  S22 TX allocat* random*  S23 S12 OR S13 OR S14 OR S15 OR S16 OR S17 OR S18 OR S19 OR S20 OR S21 OR S22  S24 S11 AND S23  S25 S11 AND S23 Limiters - Published Date: 20050101-20211231 |

| **5. Search strategy in Epistemonikos** (<https://www.epistemonikos.org/en/>) |
| --- |
|  |
| Title: (title:(Smartphone OR phone OR android OR iphone OR telemedicine) OR abstract:(Smartphone OR phone OR android OR iphone OR telemedicine)) Publication type: systematic review  Systematic review question: Interventions |
