# Supplementary material for: Smartphone-RCCT: an online repository of randomized controlled clinical trials of smartphone applications for chronic conditions
Source: Trials. 2022 Oct 27;23:909. doi: 10.1186/s13063-022-06849-x (PMC9615349; doi:10.1186/s13063-022-06849-x)
Supplement: Supplementary file 4 — Additional file 4. Codebook [file 13063_2022_6849_MOESM4_ESM.docx]

**Additional file 4. Codebook**

OSF (<https://osf.io/nxerf/>) [1]

For each study, the database has the following information (those marked with * will

be implemented in future updates of the database).

### a. Study identification

**Study_ID:** study identification number

The same study can be described in more than one article or report. Thus, we will collate all the reports for the same study under the same "study identification number". Consequently, there may be several "Record_ID" (see below) for the same "Study_ID" in the database.

**Record_ID:** record identification number

Each report will have a unique record identification number in the database.

**Source:**

1 = primary report

2= secondary report

If several reports describe the same study, we will collect the data from all them, and define the most complete and up to date article as the “primary report”. The remaining reports will be classified as “secondary report”.

**First author:** last name of the first author of the report

**Last author:** last name of the last author of the report

**Year:** publication year

**Language:** language of the report

English

German

French

Spanish

Italian

Portuguese

Other

**Reference:** citation

**Article identification number:** such as the digital object identifier (DOI) or the PubMed Identification Number (PMID).

**Incorporation_date**: date when the record was incorporated to the database.

### b. Study design

**Sequence generation:**

The study will be classified as randomized or not:

1 = Randomized study

2 = Non-randomized study: non-randomized clinical trial

3 = Non-randomized study: observational study

9 = Unclear

**Unit of allocation*:**

The study will be classified according to its unit of allocation:

1 = Allocation at the individual level

2 = Allocation at the group level (cluster design)

9 = Unclear

**Study_design_timing*:**

The study will be classified according to its timing:

1 = Parallel

2 = Factorial

3 = Crossover

4 = Stepped wedge

5 = Other

9 = Unclear

**Number_groups:** number of study arms

We will detail the number of arms in the study, for example, two, three, etc.

1 = One arm trial

2 = Two-arm trial

3 = Three-arm trial

4 = Four-arm trial

5 = Other

9 = Unclear

**IG_label:** label of the intervention group (free text)

We will allocate a label to identify the intervention group.

**CG_label:** label of control group in free text

We will allocate a label to identify the intervention group.

### c. Study setting

**Country:** country or countries where the study was carried out.

**Recruitment_invitation:** the channel/s that were used to recruit the study participants (several options can be chosen).

1 = Referral of health professional from clinical setting

2 = Website

3 = App store

4 = Social media (Facebook, Twitter, etc.)

5 = Advertisement in public (newspaper, press release, etc.)

6 = Flyer

7 = Self-referral

8 = Other (free text)

9 = Unclear

**Recruitment_clinical recruitment setting***

The setting from which the study participants were recruited.

1 = Inpatients

2 = Outpatients

3 = Both

9 = Unclear

**RITES assessment: characterization of the efficacy-effectiveness nature of trial according to the RITES tool**

We use the RITES (Rating of Included Trials on the Efficacy-effectiveness Spectrum) tool [2] to characterize each RCT on an efficacy-effectiveness continuum. We consider the following criteria:

a. Descriptor for efficacy orientation: the participants are a homogeneous population and are markedly different from those seen in usual care. Participants may be deliberately selected to comply with treatment, respond to treatment, or demonstrate the efficacy of the experimental intervention (e.g., be at high risk for the primary outcome). There may be other exclusions that would not be seen in usual care (e.g., exclusion of participants with comorbidities).

b. Descriptor for maximum effectiveness orientation: the participants are representative of the population who would receive the experimental intervention if it was part of usual care. They are similar in age, severity of illness, and comorbidities to those patients who would be candidates for the intervention in a usual care setting. They reflect diversity along parameters that could impact adherence.

We classify the efficacy-effectiveness continuum of each study according to the following categories:

1 = Strong emphasis on efficacy

2 = Rather strong emphasis on efficacy

3 = Balanced emphasis on both efficacy and effectiveness

4 = Rather strong emphasis on effectiveness

5 = Strong emphasis on effectiveness

9 = Unclear

### d. Participants characteristics

We will attempt to extract the following data of the randomized samples of the included studies.

**Sex_female_proportion*:** percentage of females

Free text

9 = Unclear

**Sex_disaggregated_data*:** availability of results disaggregated by sex group

We describe if the report presented the results disaggregated by sex:

1 = Yes

2 = No

9 = Unclear

**Gender_women_proportion*:** percentage of women

Free text

9 = Unclear

**Gender_disaggregated_data*:** availability of results disaggregated by gender

We describe if the report presented the results disaggregated by gender:

1 = Yes

2 = No

9 = Unclear

**Age*:** age of the randomised sample

We describe the age as reported (mean and standard deviation, or median and interquartile range, or other):

Free text

9 = Unclear

**Educational_level*** (free text, as reported)

Free text

9 = Unclear

**Socioeconomic_level*** (free text, as reported)

Free text

9 = Unclear

**Main_Chronic_Condition:** chronic condition addressed in the study (free text)

We preferably select the chronic condition from the list detailed in Table 1.

### e. Intervention details

**IG_Developer_Type*:** background of the developer(s) of the app (multiple responses)

1 = Non-for-profit organization

2 = University or another public research institution

3 = For-profit organization (company, start-up, etc.)

4 = Other

9 = Unclear

**IG_Developer_text*****:** name or label of the developers' organization, institution or company (free text)

**IG_Int_start*:** date, that is, month and year, when the intervention started.

**IG_framework:** reporting of a theoretical framework for the intervention.

1 = Yes

2 = No

9 = Unclear

**IG_education:** patient education

Patient education is the instruction on how to perform a behavior, information about antecedents, information about antecedents, health consequences or the salience of consequences.

0 = Absent

1 = Present

9 = Unclear

**IG_Feedback:** feedback and monitoring of the intervention

At least one of the following interventions was delivered: a) Self-monitoring of outcomes of behavior; b) Feedback on outcomes of behavior; c) Self-monitoring of behavior; d) Feedback on behavior; and e) Biofeedback.

0 = Absent

1 = Present

9 = Unclear

**IG_Goals:** goals and planning

At least one of the following interventions was delivered: a) goal setting (outcome); b) Action planning; c) Goal setting (behavior); d) Problem solving; e) Discrepancy between current behavior and goal; or f) Review behavior goals.

0 = Absent

1 = Present

9 = Unclear

**IG_Communication:** patient communication

Communication between users of the app.

0 = Absent

1 = Present

9 = Unclear

**IG_Regulation:** emotion and stress regulation

Intervention’s attempts to regulate negative emotions or reduce felt stress.

0 = Absent

1 = Present

9 = Unclear

**IG_Associations:** associations

Prompts/cues

0 = Absent

1 = Present

9 = Unclear

Reduce prompts/cues

0 = Absent

1 = Present

9 = Unclear

**IG_setting: intervention setting**

Context in which the app intervention was delivered:

1= App and face-to-face to real therapist

2 = App and virtual contact with therapist (i.e. feedback) (not live interaction)

3=App and chat with therapist via forum or other application (live interaction)

4 = App and online chat with peers

5 = App and any other interaction with health professional

6 = App only

9= Unclear

**IG_sessions:** planned dose of app in sessions.

The number of intervention sessions according to the study protocol.

1 = Minimal intervention with less than 5 sessions

2 = Moderate intervention with 6 to 10 sessions

3 = High intensity with more than 10 sessions

9 = Unclear

**IG_sessions: planned dose of app intervention in weeks**

The number of weeks during which the intervention was planned:

1 = Minimal intervention with less than 2 weeks

2 = Moderate intervention with 2 to 8 weeks

3 = High intensity intervention with more than 8 weeks

9 = Unclear

**IG_free: cost of the application to the study patients**

We describe if the smartphone app was free to the patient:

1 = Yes, free of charge

2 = No, the patient had to pay for the application

9 = Unclear

**Financial_Participants*:** financial compensation of patients for participating in the trial.

We describe if the study participants were financially compensated for participating in the trial

1 = Yes

2 = No

9 = Unclear

### g. Comparator details*

**CG_intervention: type of control condition**

We will categorize the control conditions as proposed by Gold et al. for RCTs assessing behavioral interventions [3].

**1 = Active comparator**

A treatment with an evidence-base that supports its efficacy. The comparator is different from the experimental treatment (e.g., face-to-face psychotherapy or a licensed drug)[4].

**2 = Minimal treatment control**

Treatment with fewer than four sessions [4].

**3 = Non-specific factors component control (also known as psychological placebo)**

Time with a therapist of the same duration and frequency as the experimental treatment, but no exercises or techniques regarded as therapeutic [4]. This control condition often includes educational sessions during which patients are informed only about treatments available or self-aid options. This comparator, especially if an expert guides the sessions, exhibits efficacy by its own if tested against no treatment or other interventions [4].

**4 = No-treatment control**

Contains no study treatment and is not done in a setting in which treatment would be available [5]. In the context of a RCT, patients randomly assigned to no treatment might begin to search for other forms of help [3].

The Zelen design, also known as the cohort multiple randomised controlled trial [6], is an alternative to the no-treatment control study. It requires a large sample size for a disease-monitoring study. Once the patients have been recruited, a subgroup is asked to participate in a conventional RCT, with or without a placebo or sham arm. The patients who are monitored only are used as no-treatment control [3].

**5 = Patients’ choice**

Patients can choose freely between treatments offered in a trial (e.g., one of several types of psychotherapy or between psychotherapy and medication) [3]. This type of comparator is not eligible for RCTs, but we prefer to maintain this option to follow Gold et al. classification.

**6 = Placebo pill**

A placebo pill is given to the control group, which does not receive the experimental behavioral treatment. In the context of psychotherapy research, this condition is usually part of a multi-arm trial, which also includes an arm with antidepressants as well as possible additional treatment groups (eg, psychotherapy) [3].

**7 = Specific factors component control**

Patients receive therapist time equivalent to the experimental condition, but a different or reduced number of specific factors in addition to the non-specific factors [5]. Although specific factors use a particular ingredient, such as exposure to threatening stimuli, non-specific factors are variables such as attention that apply to all interventions in question. What makes finding an adequate intervention so challenging is that behavioral interventions, for a major part, include attention as a key factor. Thus, how attention could be, or should even be, controlled for, is not always clear [3].

**8 = Treatment as usual**

Requires that the trial is done in a clinic where patients have access to some form of treatment [5]. However, the content of treatment as usual is often not reported or even assessed. Thus, treatment as usual in some trials, depending on the setting and patient population, could be no treatment at all, but in others might involve a lot of treatments (e.g., continued pharmacological treatment or behavioral treatments) that might be available as part of routine care. In our Personal View, we use treatment as usual also for trials in which the control condition is referred to as care as usual [3].

**9 = Waitlist control**

No treatment is provided during the experimental treatment period, but the experimental treatment is offered after post-treatment assessment.4 Patients are typically discouraged or even prohibited from seeking alternative treatments outside of the trial. The expectation of receiving treatment at some future point might decrease the likelihood of searching for alternative means, by contrast with treatment as usual or no or minimal treatment. Moreover, waitlist patients might experience significant nocebo effects [7], and effect sizes in trials with a waitlist control group are larger than in trials with no-treatment control conditions [3].

**99 = Unclear**

**CG_intervention_txt:** description of the control condition (free text).

### h. Outcomes

**Out_subjective health: subjective/patient reported health**

Information from patients about their own health, including functioning or disability (self-assessment).

0 = No

1 = Yes

9 = Unclear

**Out_qol:** quality of life

Information from patients about their subjective quality of life (self-assessment).

0 = No

1 = Yes

9 = Unclear

**Out_severity:**  symptom severity

Information on the severity of symptoms (self-assessment).

0 = No

1 = Yes

9 = Unclear

**Out_depression:** depression

Measurement of depression outcomes (self- or third-party assessment).

**Out_anxiety:** anxiety

Measurement of anxiety outcomes (self- or third-party assessment).

**Out_felt_stress:** felt stress

Information from patients about their subjective level of stress (self-assessment).

**Out_activity:** exercise/physical activity

Measurement of exercise/physical activity outcomes (self- or third-party assessment).

**Out_weight:** weight/diet/BMI

Measurement of weight outcomes (self- or third-party assessment).

**Out_other_behavior:** other health/risk behavior

Measurement of health/risk behavior (self- or third-party assessment), such as

sleep length or alcohol consumption which is not closely related the CC.

**Out_other_behavior_txt:** other risk behaviour (free text)

**Out_management:** measures for disease management of disorder (diabetes, hypertension, etc.)

**Out_medication:** adherence to medication

Measurement of the adherence to treatment.

**Out_motivation:** self-efficacy/self-care competence or motivation

Information from patients about their subjective level of self-efficacy, self-care competence or motivation (self-assessment).

**Out_pain:** pain

Information from patients about their subjective level of pain (self-assessment).

**Out_mindfulness:** mindfulness

Information from patients about their subjective level of mindfulness (self-assessment).

**Out_satisfaction:** satisfaction with app intervention

Information from patients about their satisfaction with the respective intervention (self-assessment).

**Out_compliance:** adherence to app intervention (attrition, drop out)

Measurement of the adherence of patients to the respective intervention

Bibliography

1. **Smartphone-RCCT Database**. In: *Open Science Framework (OSF):* [*https://osfio/nxerf/*](https://osfio/nxerf/)*.* 11 November 2020 edn. Zurich (Switzerland): Institut für komplementäre und integrative Medizin; 2020.

2. Wieland LS, Berman BM, Altman DG, Barth J, Bouter LM, D'Adamo CR, Linde K, Moher D, Mullins CD, Treweek S *et al*: **Rating of included trials on the efficacy-effectiveness spectrum: development of a new tool for systematic reviews**. *J Clin Epidemiol* 2017, **84**:95-104.

3. Gold SM, Enck P, Hasselmann H, Friede T, Hegerl U, Mohr DC, Otte C: **Control conditions for randomised trials of behavioural interventions in psychiatry: a decision framework**. *The lancet Psychiatry* 2017, **4**(9):725-732.

4. Liegl G, Plessen CY, Leitner A, Boeckle M, Pieh C: **Guided self-help interventions for irritable bowel syndrome: a systematic review and meta-analysis**. *Eur J Gastroenterol Hepatol* 2015, **27**(10):1209-1221.

5. Mohr DC, Ho J, Hart TL, Baron KG, Berendsen M, Beckner V, Cai X, Cuijpers P, Spring B, Kinsinger SW *et al*: **Control condition design and implementation features in controlled trials: a meta-analysis of trials evaluating psychotherapy for depression**. *Transl Behav Med* 2014, **4**(4):407-423.

6. Relton C, Torgerson D, O'Cathain A, Nicholl J: **Rethinking pragmatic randomised controlled trials: introducing the "cohort multiple randomised controlled trial" design**. *BMJ* 2010, **340**:c1066.

7. Furukawa TA, Noma H, Caldwell DM, Honyashiki M, Shinohara K, Imai H, Chen P, Hunot V, Churchill R: **Waiting list may be a nocebo condition in psychotherapy trials: a contribution from network meta-analysis**. *Acta psychiatrica Scandinavica* 2014, **130**(3):181-192.
