# Supplementary material for: Smartphone-RCCT: an online repository of randomized controlled clinical trials of smartphone applications for chronic conditions
Source: Trials. 2022 Oct 27;23:909. doi: 10.1186/s13063-022-06849-x (PMC9615349; doi:10.1186/s13063-022-06849-x)
Supplement: Supplementary file 5 — Additional file 5. Database structure in Open Science Framework [file 13063_2022_6849_MOESM5_ESM.docx]

**Additional file 5.** **Database structure in Open Science Framework**

Smartphone-RCCT database is available at Open Science Framework (OSF) (<https://osf.io/>). OSF is a free, open-source web application.

### 1. How to find Smartphone-RCCT in OSF

There are two ways of reaching our database in OSF.

1. Link: <https://doi.org/10.17605/OSF.IO/NXERF>
2. Using the search tool in OSF


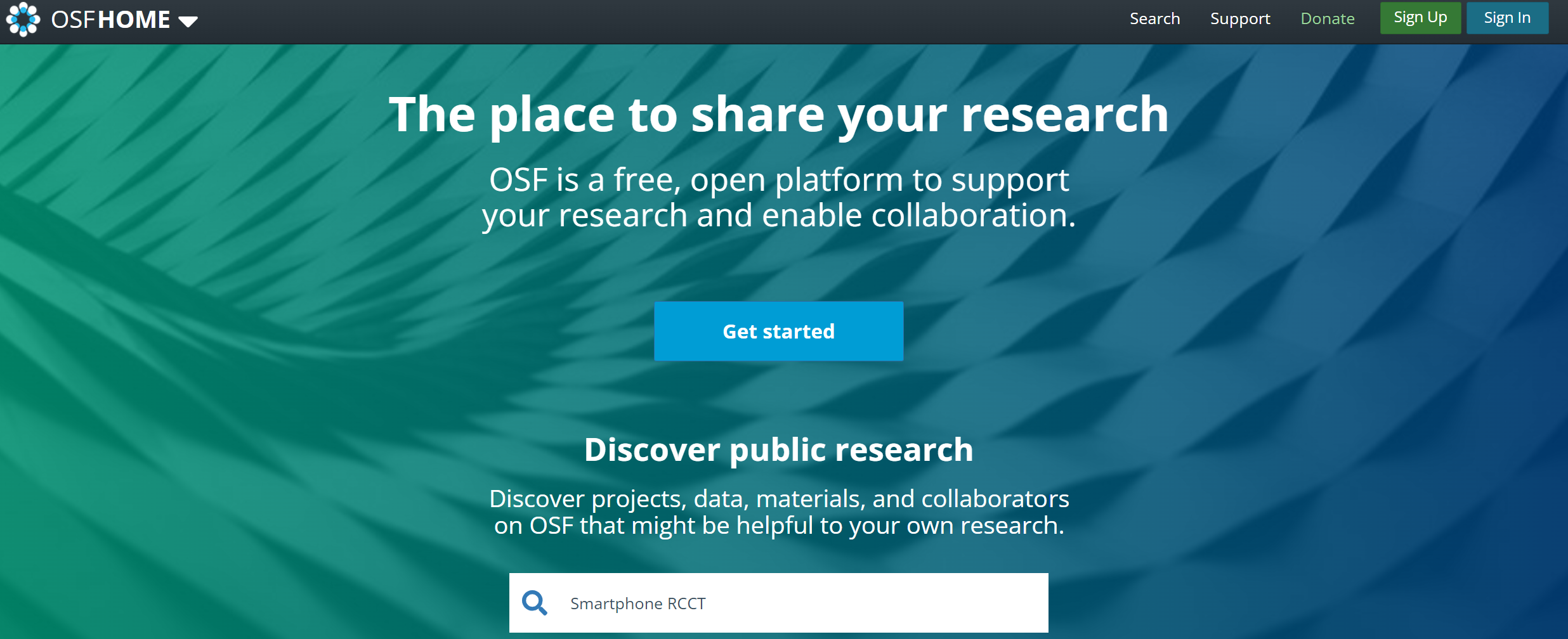


### 2. Smartphone-RCCT structure in OSF

The database has the following structure in OSF


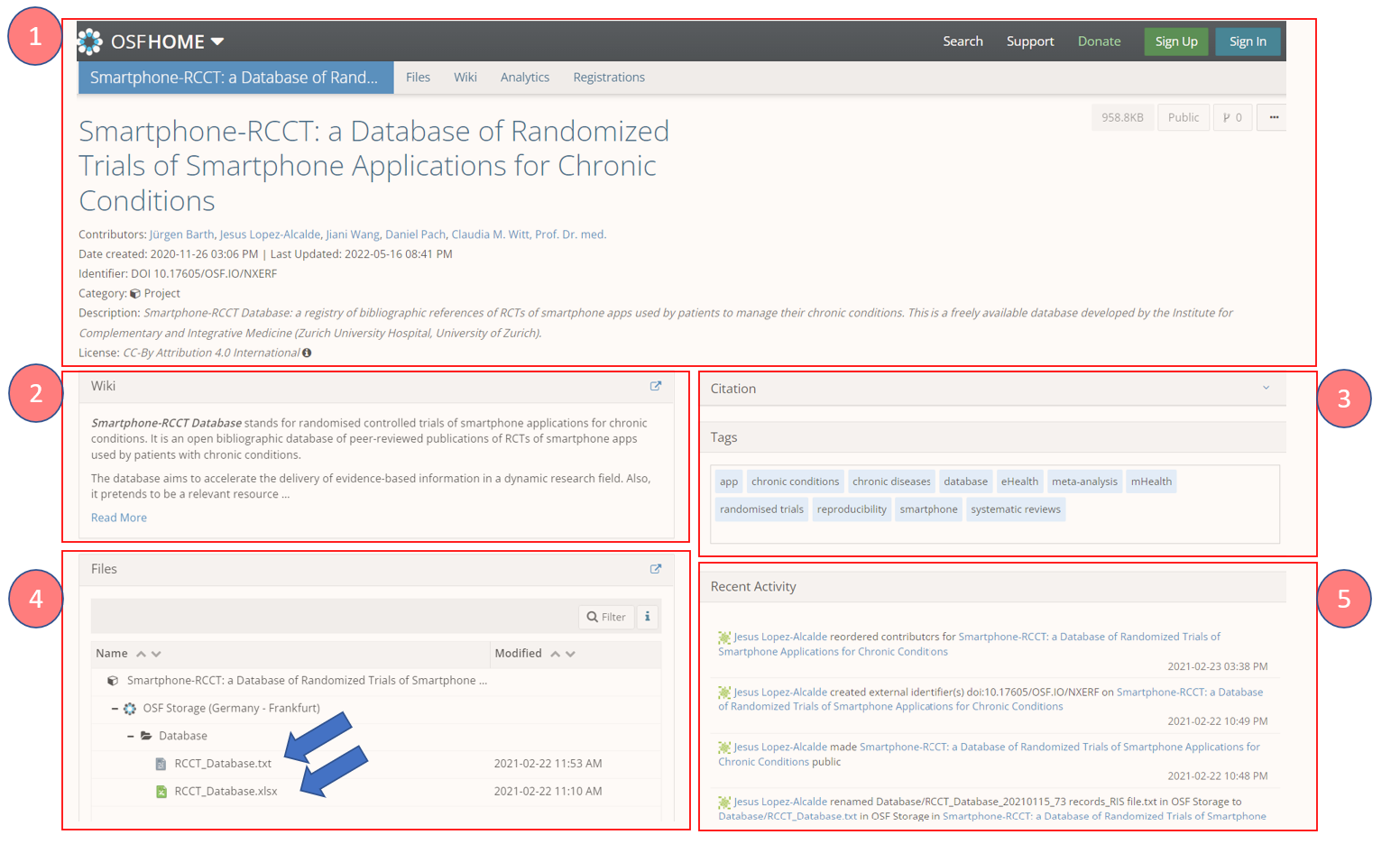


**Section 1. Database identification**

This section details the title of the database and its ID number, team, and license agreements.

**Section 2. Wiki**

This section provides a summary of the project.

**Section 3. Citation and tags**

This section provides a citation of the database and tags identifying the project.

**Section 4. Database**

This section contains the most recent version of the database in two formats.

1. RIS file ready to import to a reference management software
2. Smartphone-RCCT database in Excel format

**Section 5. Recent activity**

This section details the project activity in OSF. For example, if a new version of the Smartphone-RCCT database has been uploaded, this change will be reported here.

### 3. Smartphone-RCCT database in excel format

To access the excel database you must click on the excel file:


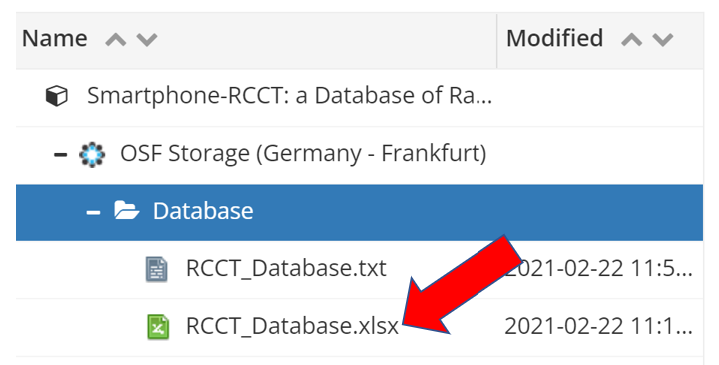


You can easily download the excel file in the next screen.


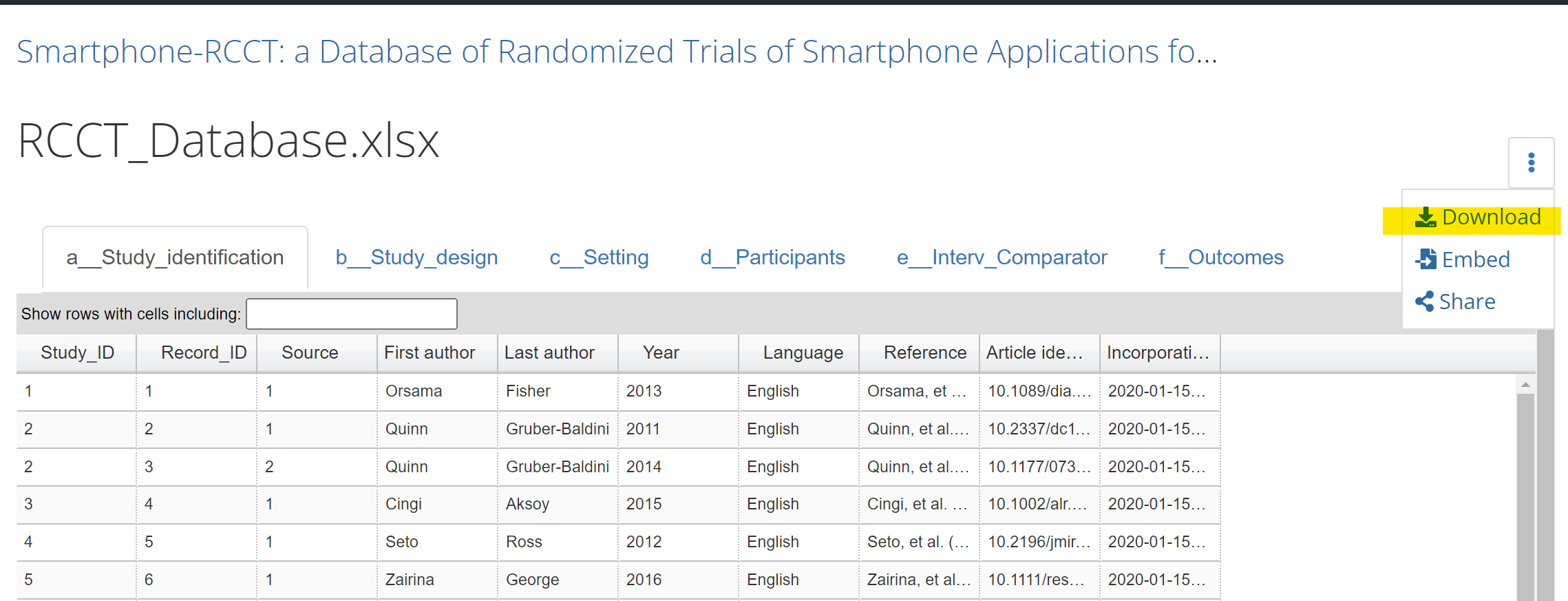


The excel contains six sheets, each of them focusing on a particular topic: a) Study identification; b) Study design; c) Setting; d) Participants; e) Intervention and comparator; f) Outcomes. Each line of the excel refers to a particular study.
